# Experiences of parents of substance-abusing young people attending support groups

**DOI:** 10.1186/0778-7367-70-11

**Published:** 2012-06-06

**Authors:** Sarah Hoeck, Guido Van Hal

**Affiliations:** 1 Department of Epidemiology and Social Medicine, University of Antwerp, Campus Drie Eiken, Universiteitsplein 1, BE-2610, Antwerp, Belgium; 2 University Scientific Institute for Drug Problems (USID), Antwerp, Belgium

**Keywords:** Substance abuse, Young adults, Parents, Support groups, Feelings of stress, General practitioners, Flanders

## Abstract

**Background:**

Substance abuse puts a burden on the physical and mental health and well-being of individuals and their families, particularly parents. Parents of substance-abusing young people are in need of professional or informal support and information. Potential and easy accessible sources are support groups. We explored the experiences of parents of substance-abusing young people attending support groups regarding several topics related to the substance-abuse of their son or daughter, the impact on their lives and their views on social support.

**Methods:**

In this small-scale qualitative study based on in-depth interviews, we interviewed parents of substance-abusing young people focusing on their experiences concerning having a substance-abusing relative and attending the support group.

**Results:**

All parents displayed feelings of stress and strain. They appeared to be highly satisfied with their participation in a support group. The expert status and knowledge of the facilitator and the provision of accurate information in the support group was also much appreciated. They were however dissatisfied by the attitude and knowledge of their GP.

**Conclusions:**

Our findings suggest that parents benefit from joining support groups, particularly in terms of emotional and social support and the practical information they received.

## Background

Substance abuse puts a burden on the physical and mental health and well-being of individuals and their families [1]. Substance abuse occurs in all age groups, but there is no doubt that young people form a vulnerable population [2,3]. While the majority of the problems surrounding adolescent substance abuse have an impact on family members, particularly parents, little is actually known about their experiences [4]. The true number of family members that are affected by the substance abuse of a relative is unknown. In 2010 in Flanders, 8500 persons were treated for substance abuse [5]. Research indicates that substance abuse by a family member has a negative impact on at least two other family members in such a way that these people need professional help [6], resulting in an estimated 17000 affected family members in Flanders. The presence of a drug abusing relative in the family affects its functioning, and may lead to high levels of stress and trauma, [7] placing all family members at risk of increased medical problems and healthcare utilisation [8]. Short-term effects on family members include increased stress, and feeling lonely, isolated, tired, unsupported, anxious, guilty, worried and confused. Longer-term effects include major changes in physical health such as ulcers or raised blood pressure and psychological health problems, including depression, behavioural disorders, panic attacks and nervous breakdowns. Furthermore, relatives have reported the deterioration of family relationships, an increased likelihood of domestic violence and a negative impact on their social life and finances [9]. Many are in need of professional or informal support and information. Potential and easily accessible sources are self-help and support groups. Those who live with and look after substance abusers are a largely hidden and isolated group; mainly due to feelings of shame and guilt [9]. Services focus primarily on the needs of the substance abuser, and not on the needs of the affected family members. Only a few studies report directly on interventions aimed at the needs of families. Since family support groups are mostly voluntary, there is little funding available for research into best practices and the effectiveness of support groups [9]. Research on the experiences and needs of family members of substance-abusing young people and support groups is therefore scarce [3,9,10]. Orford et al. [11] developed the ‘Stress strain coping support-model’ to gain a more detailed understanding of the experiences of family members living with someone misusing alcohol or other drugs. This model suggests that living with a substance abuser is stressful, the stress leads to strain, family members try to cope or respond to their situation and they experience differing levels and quality of social support [11].

The benefits of joining support groups include improved self-esteem and self-confidence, empowerment, mutual support, reassurance about the commonality of their experiences, practical information and the sharing of coping strategies [12,13]. Adverse effects of joining peer support groups may be an increase in anxiety (if people hear about problems worse than their own) and a lack of anonymity, while individual counselling can provide a more focused response and advice [13].

This small-scale qualitative study, based on in-depth interviews, was carried out to obtain detailed data on the experiences of family members of substance-abusing young people, and the way support groups meet their needs in terms of social support and the provision of information.

## Methods

### Recruitment

Inclusion criteria for family members were: having experienced long-term family disruption as a result of the drug abuse of their relative, and attending or having attended support groups. In general, family members of drug-abusing young people were difficult to reach and to persuade to participate. Eleven recognised Flemish organisations offering aid to persons with drug problems in terms of prevention, crisis management, and ambulatory and residential assistance, were at random approached for help and mediation. They are all private initiatives subsidised by the Flemish region and they occupy a crucial place in the provider network focused on the drug-abusing section of the population. Three organisations were willing to cooperate. This led to contacts with their associated support groups for family members with a substance-abusing relative. Although, only three organisations of the eleven contacted were willing to participate in the study, we are convinced that the information gathered is worthwhile. First of all, we found similar results compared to other studies on this topic. Secondly, in qualitative research it is much more important to gather opinions and attitudes to explore the field of study than to have a representative sample. Each of the support groups, which are all supervised by a trained facilitator (a psychologist or social worker) and called facilitator-led support groups, welcomes parents or other family members of substance-abusing persons and gives them the opportunity to share their experiences and feelings in open sessions. The support groups are organised weekly for 1–2 hours, and allowing participants to join or to drop out at any time.

A first hurdle to overcome was the support group policy to safeguard the anonymity of their members. However, we found a number of facilitators willing to act as a go-between and we were eventually permitted to attend some group sessions in order to invite the family members to participate in the study. This procedure resulted in 12 out of 48 family members, all parents, agreeing to take part in the study. Since parents in these support groups take part anonymously and the contacts with the parents took place by means of a mediator, information concerning the non-respondents is unfortunately lacking.

### Interviews

**Figure 1:**
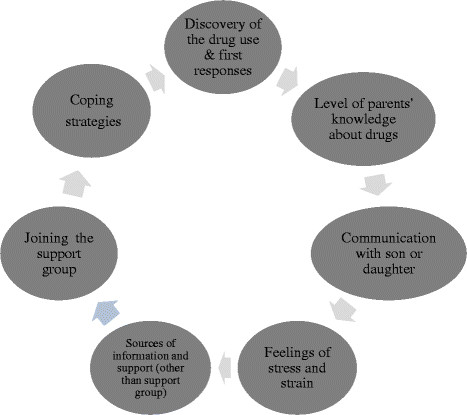
Key themes used for the guidance of the interview.

Semi structured in-depth interviews allow participants to describe their experiences and perspectives, and to reflect upon their responses through interaction with the interviewer. A list of previously fixed key themes was used to guide the interview (Figure 1) and focused mainly on the experience of having a substance-using family member and on their attendance at the support group. The order of the topics which were discussed depended on the spontaneous flow of the interview. When necessary, additional questions were formulated to gain more in-depth information. The risk of participants responding in a way that they felt was socially acceptable, rather than honest, was reduced by the researcher actively seeking to reassure the participant and establish a trusting relationship. Most interviews were conducted at the home of the parent. All interviews were taped and transcribed in full. The interviewer (SH) was professionally trained both at applied and academic level, and had extensive experience with conducting in-depth interviews in a social context. The average duration of the interviews was approximately 100 minutes.

### Ethical aspects

All participating parents gave oral informed consent to the mediator and explicitly confirmed this to the interviewer before the start of the interview. It was clearly agreed by all involved parties that the anonymity of the parents and their drug-abusing sons or daughters would be guaranteed at all times. At any time, parents could indicate that they did not want to continue the interview. The participants’ anonymity was ensured throughout the study. No personal information about the participants was passed from the interviewer to other people involved in the study and information regarding identification was not transcribed. No monetary or other compensation was given.

### Data analysis

We analysed the interviews using thematic analysis and the principles of the grounded theory approach [14], to identify any recurring themes across the parents’ experiences [15,16]. All the transcribed interviews were read several times to gain a holistic view of the experiences and viewpoints of the parents. A highlighting approach was used to identify the essential themes and patterns, which were clustered into core themes. This procedure was independently performed by two investigators (SH and GVH).

## Results

### Characteristics of the substance-abusing young persons

A number of characteristics of the young people whose parents were interviewed are summarised in Table 1. All but one (a young man aged 17) were over 18 years of age and should be classified as ‘young adults’ following the terminology of the European Monitoring Centre for Drugs and Drug Addiction (EMCDDA), which uses the range 15–34 years [2]. The age range of the drug-abusing young adults was 17–34 years, with a mean age of 25 years. All but one were males and seven of them were no longer living with their parents at the time of the interview. The average duration of their drug abuse was nine years, with a range of 4–13 years. At the time of their parents’ participation in a support group or before, all of the young people were abusers of illicit substances including cannabis, cocaine, amphetamines, and heroin. More than half of them (7/12) had been in contact with the police or had been brought to justice for various reasons, including theft, possession of drugs and dealing; in some cases resulting in enforced counselling, treatment or imprisonment.

**Table 1:** Characteristics of the young adults whose parents were interviewed

No.*	**Gender**	**Age**	**Living with parents**	Duration of abuse**	Current abuse**	Drugs**	**Contact with police**
1	F	20 y	no^a^	6 y	†^c^	polydrug abuse, with heroin	yes
2	M	20 y	yes	7 y	yes	cannabis, amphetamines	no
3	M	23 y	no^a^	9 y	yes	cannabis, cocaine	yes
4	M	27 y	no^a^	13 y	no^d^	polydrug abuse, with heroin	no
5	M	27 y	no^a^	11 y	yes	amphetamines	yes
6	M	24 y	yes	8 y	-^e^	cannabis	no
7	M	27 y	no^a^	13 y	yes	polydrug abuse, no heroin	yes
8	M	17 y	yes^b^	4 y	yes	cannabis	no
9	M	34 y	no^a^	12 y	no^f^	cocaine	no
10	M	28 y	yes	7 y	yes	polydrug abuse, no heroin	yes
11	M	27 y	yes	12 y	yes	polydrug abuse, no heroin	yes
12	M	28 y	no^a^	10 y	yes	polydrug abuse, no heroin	yes

* Corresponds with numbering in Table 2.

** Drug abuse as perceived by the parent at the time of the interview.

^a^Not living with parents during the last years of drug abuse.

^b^Living with his father during the weekends and during the week with his mother.

^c^Daughter was murdered.

^d^Drug abuse ended one year before the interview.

^e^Receiving treatment at the time of the interview.

^f^Drug abuse ended two years before the interview.

Flanders, Belgium, 2005-2006.

These young men and women had been abusing illicit drugs for a minimum of four years and were showing problematic behaviour as a result of their abuse. Moreover, all of the interviewed parents suspected their son or daughter of being a multiple abuser of illicit drugs, and an abuser of alcohol and tranquillisers at the same time.

### Characteristics of the interviewed parents

It was not easy to convince parents to take part in an in-depth interview. Most of the support group members approached who refused to participate were unwilling to do so for emotional reasons. In their view, support group participation required a great deal of effort and they found it difficult to talk about their personal experiences even in that context. Accordingly, the most often quoted reason for non-participation was the high emotional level of the information needed. Feelings of shame and guilt, and the need for protection of personal privacy, represented additional barriers. Those parents that were selected, however, cooperated in full.

The characteristics of the interviewed parents are displayed in Table 2. The majority of them (9/12) were women. Two thirds of the group were married and one third was divorced. The average number of children in these families was 1.8. Parents had been dealing with the problematic situation for an average of 8.5 years. The duration of the parents’ participation in the support group varied between a couple of sessions to weekly participation over several months and more (Table 2). Participating fathers usually appeared to be more interested in judicial and practical aspects of the problem than mothers.

**Table 2:** Characteristics of the interviewed parents

No.*	**Gender**	**Civil status**	**Number of children**	**Employment status participant**	**Employment status (ex-) partner**	**Participation in support group**
1	F	married	2	teacher	self-employed	2 y
2	F	divorced	2	employee	self-employed	2 sessions
3	F	married	3	housewife	employee	> 5 y^d^
4	F	divorced	2	employee	executive	> 5 y^d^
5	F	married	1	housewife	worker	> 5 y
6	F	divorced	2	housewife^a^	self-employed	6 sessions
7	F	married	2	employee	self-employed	5 sessions
8	M	divorced	1	worker^b^	worker	6 sessions
9	M	married	3	retired^c^	employee	> 2 y^d^
10	M	married	1	worker	housewife	< 1 y
11	F	married	1	employee	worker	6 sessions
12	F	married	2	worker	worker	< 1 y

* Corresponds with numbering in Table 1.

^a^ With a university degree.

^b^ Foreman.

^c^ Retired professor.

^d^ Currently a voluntary worker in a support group.

Flanders, Belgium, 2005-2006.

### Discovery of the drug use of their son or daughter

Before joining the support group, parents were often not able to link the physical and behavioural changes their child underwent with possible drug abuse. For example, when they noticed mood swings, outbursts of anger, changes in their son’s or daughter’s circle of friends or school record, or an increased need for money, they all first thought these facts were related to puberty. Only after they had actually joined the support group and received specific information about substance abuse did they see a connection between the observed changes and the substance abuse. However, most (9/12) of the parents mentioned behavioural changes as the first sign of possible drug use. Half of the parents were informed by a third party (police or hospital) about the drug use of their son or daughter, three parents were informed by the drug abuser him/herself, one parent found drugs in the room of his son and only two parents actually linked behavioural/physical changes to drug abuse and made their child confess.

Parent 5: ‘His behaviour changed at the age of 14. He skipped school, neglected his friends. But it was not until the police came for him we really knew he was into drugs.’

Parent 11: ‘We never saw him. He was always late at home, not communicating with us, isolated himself in his room. We thought it was his age.’

Parent 12: He lost some weight, but he always was very skinny, so at first we didn’t make much of it. Some years later I found drugs in his room. He was 21. At the time I didn’t know what it was, now I know it must have been cocaine.’

### Level of the parents’ knowledge about drugs

Most (10/12) interviewed parents’ knowledge about drugs and drug abuse was very limited before they joined the support group. Only a few of them (2/12) had provided some type of information to their sons or daughters before the abuse came to light; these parents had previously faced alcohol abuse in their own environment. Most of the parents stated that they never thought drug abuse would apply to their own family and therefore they did not feel the need to become better informed or to inform their sons or daughters about the risks and dangers.

Parent 5: ‘Actually, I never heard anything about illicit drugs. As a result, I never developed an opinion of my own on the subject.’

Parent 12: ‘I knew nothing about drugs. Absolutely nothing. We did not know what it was and barely ever heard of it. Who would have thought this would ever apply to us?’

### Communication with son or daughter

Almost all parents indicated that there was no appreciable open communication in their family about feelings such as love, aggression, anger, jealousy, and other emotionally charged themes. Everyday conversations only involved unimportant issues. As a consequence, drug-related problems never came up.

Parent 7: ‘It was simply impossible to talk with our son about anything. We discussed only superficial matters.’

A majority (8/12) said that they did not hold an opinion or view on substance abuse before their son or daughter showed visible signs of use and they attributed this to their lack of experience with drugs in their own youth. The young people were generally (11/12) not prepared to talk about the problem (or about any other important issue) with their parents. No matter how difficult communication proved to be on both sides, all interviewed parents kept trying, in vain, to identify the problem and to convince their son or daughter to seek professional help.

Parent 4: ‘They are not open to argument. According to my drug-abusing son, there is no problem at all and everything is under control. If you get the discussion going, they simply run off.’

The resulting feeling of powerlessness, combined with a sense of being let down by their general practitioner (GP), were important elements in the parents’ subsequent search for help, leading to their participation in the support group.

### Sources of information and support (other than support group)

The moment the parents were informed about the drug abuse, the majority of them (10/12) consulted their GP for help and to gather specific information about drugs. Only one parent, however, was moderately happy with the information and advice received. The vast majority (11/12) thought their GP seriously underestimated the problem and thought he or she inadequately referred them to specialist help. They were very dissatisfied with the interaction with their GP and were of the opinion that their GP was not well-informed about drugs and drug abuse.

Parent 5: ‘Our GP did not really take the matter seriously. He had no idea to whom he had to refer us. We did not experience this as problematic in the medical sense, but it was a real problem with regard to support and referral.’

Parent 6: ‘My son went to see my ex-husband’s GP. This man was unable to assess the situation correctly. He compared it with giving up smoking. The boy had to take the initiative and was supposed to give up his drug abuse on his own. This GP was clearly unaware of the gravity of the situation.’

Parent 10: ‘We went to our GP right at the beginning, shortly after we had discovered these pills in the room of our son. He asked where he went out. He said that XTC use was an integral part of the current music culture. It would pass automatically. My wife and I both felt quite uneasy about this.’

Parent 11: ‘He said that parents often react in panic when they find drugs in the rooms of their children. I told him that we did not find soft drugs, but a white powder instead. But even then he said there was no need to blow the problem up.’

Two parents first contacted organisations specialising in assistance for problematic drug abusers, but only after their son or daughter got into trouble and was required to get treatment. The other parents (10/12) also contacted specialist health workers, but after they had contacted their GP. All parents were dissatisfied with these contacts because these professionals were unwilling to release information in order to protect the drug abusers’ confidentiality. Although they were dissatisfied about the information they received about their son or daughter's treatment, they accepted the professionals’ advice to participate in an associated support group.

Parent 1: ‘I regret that they wouldn’t talk about the conversations they had with my daughter. Did they know how well she could manipulate?’

Parent 2: ‘We really went away empty-handed as regards information about his situation. They couldn’t even tell us if he showed up at the obligatory meetings or not.’

When asked which their main source of information was, the parents almost unanimously mentioned their support group. Only one participant reported having extensively gathered information shortly after the discovery of the drug abuse, by means of research using the library and the internet.

### Feelings of stress

The core emotions that parents had to deal with were feeling worried and anxious (12/12), helpless and despairing (10/12), low and depressed (11/12). Most parents also mentioned feeling guilty (9/12). Furthermore, parents’ self-image and self-confidence were undermined by their experiences (10/12). Most parents (10/12) mentioned some form of aggressiveness, irritability, verbal abuse, rudeness, domineering behaviour, threats. Five parents (5/12) were victims of physical violence such as pushing, punching and hitting. All parents experienced feelings of uncertainty and had, in general, an imperfect knowledge of what was going on. Uncertainty because of unreliability of the child’s presence in the home, their comings and goings, being absent when they were expected to be home, arriving home at uncertain times and in uncertain states. All the parents were worried about their child, not only about the drug abuse itself and about the people they associated with, but also about their physical and mental health, their financial affairs, their safety, their education or work.

Parent 4: ‘When they told us in the hospital which kind of drugs he was using, our world stood still. We were anxious and didn’t know what to do.’

Parent 12: ‘From the beginning I was really anxious, scared and worried about what could happen to him. The fear never went away. Even now when he no longer lives with us, I still can’t sleep and I am worried sick.’

Parent 6: ‘I’ve brought him up. What have I done wrong?’

Parent 12: ‘I had to stand between my son and my husband. I was afraid he was going to kill my husband.’

Parent 4: ‘He became more and more aggressive and we really were afraid of what he would do to us.’

All parents indicated that home and family life were threatened by the drug abuse of their son or daughter. Seven of the eight married parents mentioned an enormous pressure on their relationship and on family functioning in general. The majority of the parents (7/12) disagreed about how to handle the situation, in most cases the father seemed to keep some distance at an earlier stage than the mother. The majority of the parents with more than one child (6/8) mentioned a disturbed relationship with the brother or sister of the drug abusing child.

Parent 5: Especially now with the cold weather, I let him in at night, against the advice of my husband. During one night he started a fire, another night he attacked me and my husband. Still, again and again I let him in.’

Parent 7: ‘We almost separated because of our son. You can’t imagine the impact of such a situation on your relationship. Especially because I just wouldn’t let go, while my husband really tried to take some distance.’

Parent 11: ‘Based on facts I knew I had to throw him out of the house, but I couldn’t. My husband doesn’t let him in at night anymore.’

Parent 1: ‘My oldest daughter once said ‘I’d better start using as well, then I might get some attention’.

Parent 7: ‘At one moment, my other son really cried out for help. He said we were always arguing, and that everything revolved around X. That was true, but then we were not aware of it.’

Parent 1: There were often conflicts about money and possessions. I used to sleep with my wallet and jewels under my pillow. Things went missing all the time.’

### Joining support group

The parents did not value their support group only as a source of information, but equally and empathetically perceived it as a means to boost their morale. This was confirmed by all participants but one. Half of them gained courage from happily ending stories shared with them by other group members. For these reasons, almost all interviewees were satisfied with their participation in the group and considered its role as crucially important. They stressed that the most important benefits of joining the support group were support, an increased understanding of their needs, an increased focus on themselves, and an increased confidence to withdraw from the situation. The expert status and knowledge about addiction and treatment of the social worker leading the groups satisfied the need for information expressed by all parents.

Many of them had to cross an emotional threshold before they actually took part, but gradually learnt to overcome their feelings of guilt, shame, and failure – which were more prominent for those who had only one child. Furthermore, they felt the group taught them how to distance themselves from their problematic situation and how to deal with their risks and fears.

Parent 3: ‘Sharing your story with others not only makes you feel good. It also makes you feel you are helping someone else. We give each other good advice and make suggestions. But the most important thing is to get support and understanding.’

Parent 4: ‘Once you have crossed this threshold, it is quite a relief to talk with people that feel the same as you do. In this group you get the feeling that there is a chance that you will get out of the problem. You also hear positive stories and this offers hope.’

Parent 8: ‘You get a sense of helping people by telling your own story. And that’s such a nice feeling. You also hear other stories and you learn from them. You find out that you are not alone with all your questions.’

Parent 10: ‘It makes me feel good to hear that I am not the only one to face such a situation. You hear about recognisable events and problems. Some parents make useful suggestions or simply give support and this is very important to me.’

Parent 12: ‘It made me realize that I’ve got a life of my own. I felt stronger and a bit less depressed and anxious.’

### Coping strategies

At first most parents tried to limit the drug abuse by making some rules about it, trying to regain control and punishing their son or daughter (9/12), but they quickly realised that it was no use. Some parents even tried to cover up the drug abuse, gave money to prevent crime or were too frightened to do anything. After joining the support group most parents became more able to distance themselves from the situation. In the beginning, 4 out of 12 parents tried to reconcile themselves with the drug abuse, mostly thinking it was just temporary (4/12). The majority of the parents (9/12) indicated it was important to introduce some distance, physically, emotionally or both. Most parents (8/12) talked about the importance of trying to live with the anxiety and fear.

Parent 7: ‘We tried to punish him for his behaviour, the lies, the stealing, but we didn’t know how. He just never listened. It just became worse.’

Parent 4: ‘We have given the fact that something bad could happen to him a place. Day and night we worried sick about him, wondering what could happen each night.

Parent 5: ‘At a given point you just have to let go and you have to accept that bad things could happen to him and there is nothing you can do about it. You have to learn to live with it.’

The main results of the study are summarized in Table 3.

**Table 3:** Main results of the study

**Discovery of the drug use**	**Parents’ knowledge about drugs/communication**	**Sources of information and support**	**Feelings of stress**	**Joining the support group and coping strategies**
1. Informed by a third party (police or hospital)	Very limited knowledge before joining the support group.	1. GP	Worried and anxious	Information about addiction and treatment
2. Informed by the abuser	No opinion or view on substance abuse before the discovery of the drug abuse of their son or daughter.	Con: underestimation of the problem, inadequately referred them to specialist help, not well- informed about drugs	Low and depressed	Boost their morale
3. Parents themselves	No open communication about drugs and other difficult themes.	2. Specialized organizations	Uncertainty	Support, understanding of their needs
		Con: confidentiality hinders follow-up of substance abusing child	Family functioning threatened	Learning to distance themselves from the problematic situation
		Pro: advice to participate in support group	Married parents: strain on their relationship	Overcome their feelings of guilt, shame and failure
		3. Support group	Parents with more than one child: disturbed relationship with brother/sister of drug abusing child	Learn to deal with risks and fears
			Helpless and despairing	Coping strategies:
			Self-image and self-confidence undermined	- Important to introduce some distance, physically, emotionally or both
			Victim of aggressiveness, verbal abuse, threats	- Important to learn to live with the anxiety and fear
			Feeling guilty	
			Disagreement about handling the situation between parents	
			Being victim of physical violence	

## Discussion

The social support model indicates that social relationships have beneficial effects on physical and psychological health and well-being [17,18]. Interaction within a support group represents a framework in which shared experiences, an increased understanding of their needs and a focus on themselves favour participants’ health outcomes. The knowledge about addiction, its treatment and prognosis, of the social worker leading the support group can be used to motivate the group, and helps the group to work in a constructive way, aiming to manage stress by recognising feelings and gaining insight into the situation [19].

Our research has its limitations. The first of these concerns the small group of parents interviewed. The conclusions are, however, not necessarily meant for extrapolation to the entire population of parents of substance-abusing young adults. Parents were recruited from the support groups, and may therefore not be representative of all parents of drug-abusing young people. These parents could be particularly active in their efforts to cope with the drug abuse of their son or daughter. However, by the end of the series of interviews, the researcher felt that no additional insights were to be obtained by interviewing more parents, i.e., theoretical saturation had been reached [15]. Secondly, the majority of the participants were women, which is a general characteristic of support groups. In line with findings in other research, men are less likely to seek psycho-social support in general [20,21] and support with regard to drug problems of family members in particular [9]. A female approach is most valuable, but does not necessarily cover the whole spectrum of possibilities to deal with these specific problems. A third limitation is partly linked with the previous one. Since the impact of drug abuse on family life is usually high [22], the voice of the second parent and even of the siblings and other family members of the drug-abusing young man or woman should ideally also be heard, while only parents participated in this study. Lareau (2000) [23] indicated that in the case where only one family member can be interviewed, mothers are a good choice because they are ‘core family members’. And, finally, a fourth limitation is associated with the way the type of drug abuse was assessed. It was only reported by the involved parent and not, for example, by the young adult him or herself, or by other family members. This potentially makes these findings to somewhat less certain.

A review of literature data showed that the information available to describe who is likely to participate in support groups of all kinds is not comprehensive [12,24]. Some authors suggest that attendees may be more motivated to change and are socially more competent than non-attendees [25]. Next, in the present study only some of those who were active in a support group were willing to answer questions. As a result, this study dealt with a selection with special characteristics. Three of the twelve participants were currently working as voluntary workers in the support group and could therefore not be seen as representative for other group members. Nevertheless, our sub-population displayed interesting features and provided useful information. Many responses were remarkably consistent. Moreover, representativeness is not the main aim when conducting qualitative research.

The over-representation of women in our study population remains a factor of interest. The fact that women pronouncedly outnumbered men, both in their participation in the contacted peer support groups and in the population of interviewees, corresponds with many of these women’s opinion that their male partners usually take a more rational and more radical stand regarding their son or daughter. In their view, mothers keep on trying to help their drug-abusing child whatever it may take, while fathers, from a certain stage, judge it more appropriate to distance themselves. Literature data show that the role of families, and especially of mothers, is a most important one in regulating the drug-related behaviour of young people [26].

Equally, the great majority of the drug-abusing children were male, which may be regarded as a reflection of the general situation. The abuse of illicit drugs and alcohol is primarily a male problem, as confirmed by many studies [2,27,28].

Participating parents were not aware of their son or daughter abusing other drugs apart from the four classical categories (cannabis, cocaine, amphetamines, and opioids such as heroin) and alcohol. Specific designer drugs, inhalants, non-prescribed sedatives, sleeping pills or anxiolytics, and hallucinogens such as mushrooms, mushroom- or plant-derived substances, and LSD were not mentioned. Typically, participating parents were usually not well informed about drugs in general and the risks and resulting problems. As a consequence, they felt insecure about their competence to counsel their son or daughter, either before or after the discovery of the drug abuse. Together with this is the fact that basic problems are not brought up for discussion within the family. The question arises as to whether this lack of communication is more pronounced in families with drug-abusing children than in others. A number of studies clearly revealed a link between parent–child connectedness (including the ability to talk about problems) and behavioural health indicators (including risk behaviour such as substance abuse), both for young men and women [29,30].

One of the major findings of this inquiry is that the interviewed parents were highly satisfied with the positive aspects of their participation in the support group in terms of understanding, sympathy, comfort, information, and support they received. Their participation had a therapeutic effect and helped them to overcome negative feelings regarding their son’s or daughter’s drug problem and the information other parents and the social worker gave them in general. Also, they considered it as a reassuring thought that other families face comparable problems and that they could get help based on the experiences of the group. Most of them realised that communication with their child and peers ranks high among the means they have to cope with the situation. Most parents would have recommended others to join a support group.

Seeking help is often a difficult process. Marshal [13] reported that many family members try to cope on their own for a long time before they look for help, and that they feel ashamed when they do so. Family members are reluctant to open the problem up to anyone other than those living in the immediate household. The reluctance to seek support is related to feelings about what it means to be a good parent and the shame that the parent might feel if it was known outside the family [31].

The interviewees generally joined up with a group after having appealed to a primary healthcare worker for information and help, and having gone through a frustrating disappointment. In their opinion, their GP usually was not well informed regarding drugs and seriously underestimated both the problem of their son or daughter and its impact on their family situation. They were not referred to or informed about specialised care. This finding is concordant with the results of Dutch research in which one third to one half of the parents participating in support groups made the decision to join because they were discontented about the role of the primary healthcare provider [32]. It is contradictory to the widespread view that primary healthcare providers such as family physicians are the most likely and credible source of professional advice and assistance for a wide range of health issues, including substance abuse [33-35]. Family physicians, however, are typically not well trained in these domains. In a postal survey carried out among GPs in the United Kingdom regarding barriers to brief alcohol interventions, respondents primarily mentioned insufficient time and training [36]. In a qualitative study by McKeown *et al*. (2003) [37], a majority of interviewed GPs felt they had insufficient knowledge of substance abuse, due to a lack of training or experience. On the other hand, it has been shown that expansion of GP training and care protocols sharply improve guidance and management of, for instance, alcohol-dependent patients [38]. Professional people are not always as helpful as might be hoped, which left family members feeling that they had received inadequate information or support, and social workers are often unable to provide information due to a need to respect the drug abusing relative’s confidentiality [31]. The overall conclusion about social support is that good social support, when it occurs, is highly valued, but that, unfortunately, the kind of social support that family members need is very often denied to them [31]. Research indicates that family members with good social support are more able to give support to their drug abusing relatives and to cope with the situation [39].

## Conclusions

This selected group of parents spoke frankly about their experiences while trying to come to terms with their problem. There was no doubt that social support is highly valued by the parents. Simply having someone to talk to and who listened to the parent, in an atmosphere of acceptance and support was crucial. The provision of accurate information by the facilitator was also much appreciated. The positive role of support groups is only to be welcomed. Both the attitude and knowledge of the GP’s, however, deserve special attention [40]. Parents in our study displayed many of the expected signs of stress and strain and were employing a variety of typical coping strategies to try to respond to their situation, as described by the SSCS (stress strain coping support model) model [11]. This model suggests that support and coping must change first, and that a linked reduction in stress and strain can follow. Although there is still much to be learned, our study indicates that a core set of experiences of family members of drug-abusing relatives exists. These findings suggest that parents benefit from joining support groups, particularly in terms of the emotional and social support they receive from their peers, the practical information they obtain and the changes in coping mechanisms they can make.

## Competing interests

The authors declare that they have no competing interests.

## Authors’ contribution

SH and GVH developed the design of the study. SH conducted the interviews and transcribed them in full. SH and GVH independently identified the essential themes in the interviews. SH reviewed the literature and drafted the manuscript. SH and GVH critically revised the manuscript. Both authors have read and approved the final manuscript.
